# Hypertrophic Cardiomyopathy due to Mitochondrial Disease: Prenatal Diagnosis, Management, and Outcome

**DOI:** 10.1155/2013/472356

**Published:** 2013-01-03

**Authors:** Lutgardo García-Díaz, Félix Coserria, Guillermo Antiñolo

**Affiliations:** ^1^Unidad de Gestión Clínica de Genética, Reproducción y Medicina Fetal, Instituto de Biomedicina de Sevilla (IBIS), Hospital Universitario Virgen del Rocío, CSIC, Universidad de Sevilla, 41013 Sevilla, Spain; ^2^Unidad de Gestión Clínica de Pediatría, Sección de Cardiología Infantil, Hospital Infantil, Hospital Universitario Virgen del Rocío, 41013 Sevilla, Spain; ^3^Centro de Investigación Biomédica en Red de Enfermedades Raras (CIBERER), Hospital Universitario Virgen del Rocío, Avenida Manuel Siurot s/n, 41013 Sevilla, Spain

## Abstract

A case of prenatally diagnosed fetal hypertrophic cardiomyopathy is reported. The mother was referred to our department at 37 weeks' gestation because of suspected congenital heart disease. Prenatal echocardiography showed biventricular hypertrophy and pericardial effusion, without additional abnormalities. Postnatal echocardiography confirmed prenatal diagnosis. Neonatal EKG showed biventricular hypertrophy and Wolff-Parkinson-White syndrome. Skeletal muscle biopsy was consistent with mitochondrial oxidative phosphorylation defect involving a combined defect of respiratory complexes I and IV. Echocardiographic followup during the first year of life showed progressive regression of hypertrophy and evolution to left ventricular myocardial noncompaction.

## 1. Introduction

Cardiomyopathies are among the clinical presentations associated with mitochondrial oxidative phosphorylation defects [1]. Cardiomyopathies are a significant clinical problem associated with sudden death, which can be isolated or associated to other cardiac and noncardiac conditions [1].

Neonatal mitochondrial cardiomyopathies are often characterized by hypertrophy of one, generally left, or both ventricles. Ventricular dysfunction may be progressive in-utero and after birth. Neonatal cardiomyopathy often lead to fatal heart failure, although may eventually progress into a dilated form [2], into noncompaction of left ventricle, improve or even regress completely [3].

We present the case of a fetus diagnosed with hypertrophic cardiomyopathy at 37 weeks' gestation, which was finally found to have a mitochondrial oxidative phosphorylation defect.

## 2. Case Report

**Figure 1:**
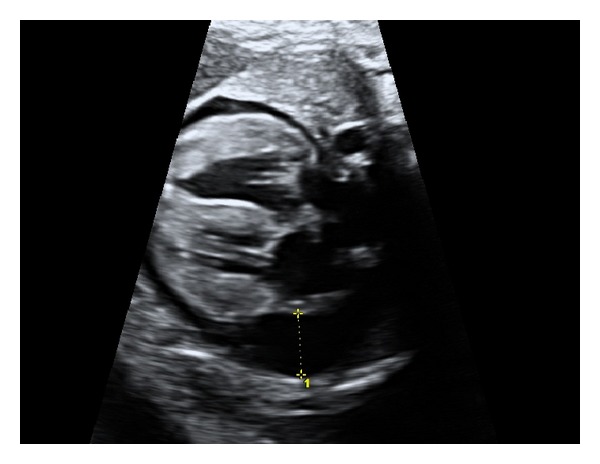
Fetal echocardiography at 37 weeks shows cardiac hypertrophic involving the right ventricular, left ventricular and interventricular septum, and pericardial effusion.

**Figure 2:**
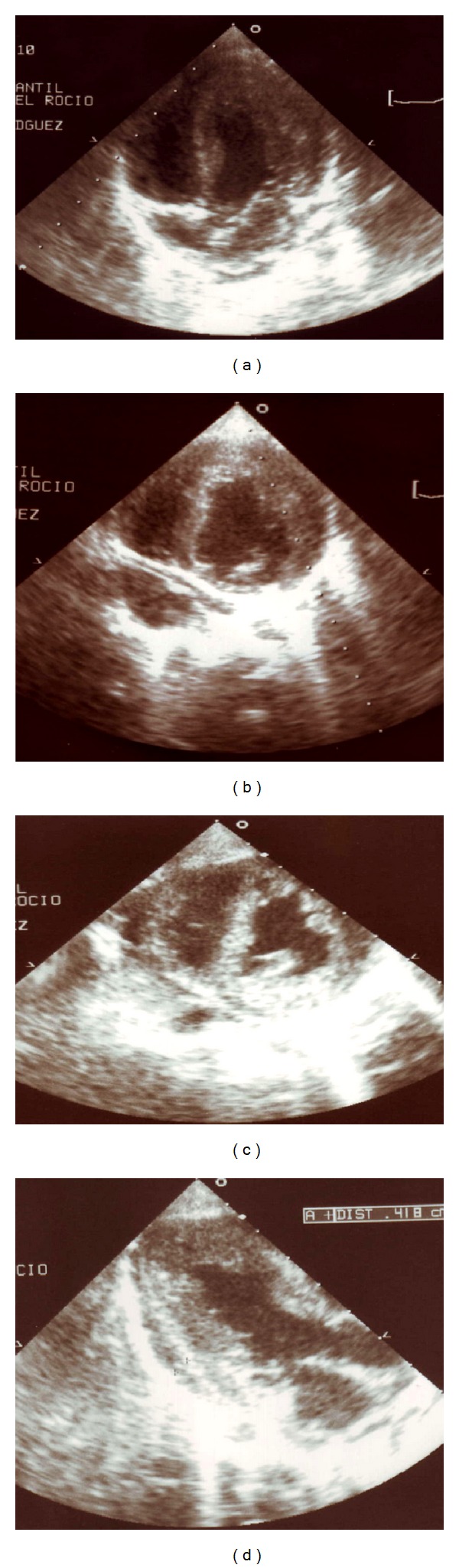
Neonatal echocardiography showing cardiac morphology similar to that in fetal echocardiography.

**Figure 3:**
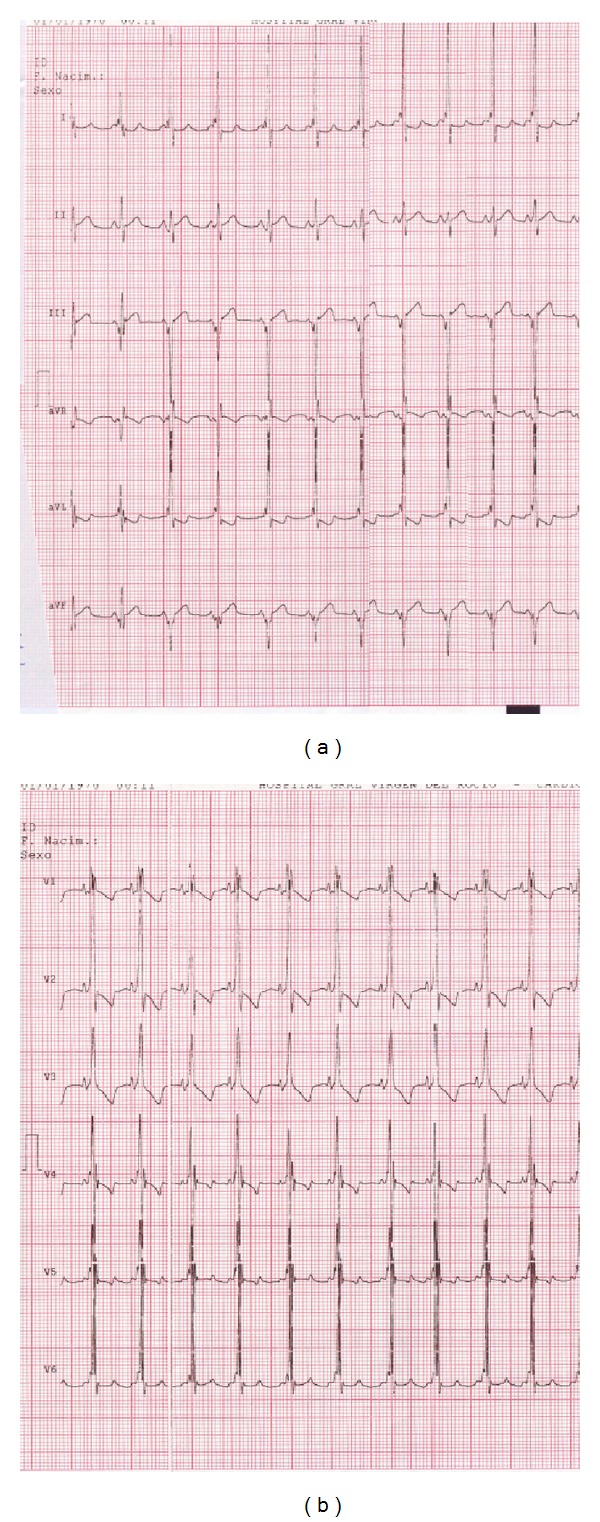
Neonatal electrocardiogram showing biventricular hypertrophy.

A 30-year-old woman, gravida 3, was referred to our Department at 37 weeks' gestation due to an abnormal obstetric ultrasound. Personal and pregnancy history were unremarkable. Familial history revealed a previous child died because of congenital cardiac anomaly, namely double outlet right ventricle. Fetal echocardiographic evaluation showed biventricular hypertrophy and pericardial effusion (see Figure 1), and left ventricular ejection fraction was 55%. The fetus showed no other sign of cardiac failure or additional heart abnormalities. Fetal ultrasound examination revealed an otherwise healthy male fetus. At 38 weeks' gestation, a 2,836 g male was born by elective caesarean section. Apgar score was 10 at 1 minute and 10 at 5 minutes. The neonatal echocardiogram confirmed fetal diagnosis (see Figure 2) and left ventricular ejection fraction was 52%. Electrocardiogram (EKG) showed biventricular hypertrophy with preexcitation, which was consistent with Wolff-Parkinson-White syndrome (see Figure 3). Chest X-ray showed moderate cardiomegaly. Treatment was started with diuretics and digoxin and the patient remained stable, without cardiac symptoms or arrhythmia.

Skeletal muscle histopathological and spectrophotometric studies were diagnostic of a mitochondrial oxidative phosphorylation defect involving respiratory complexes I and IV.

Follow-up echocardiography showed progressive regression of hypertrophy, except at left posterior wall and interventricular septum, with no changes in ventricular function and no pericardial effusion. At the same time, left ventricular myocardium developed trabeculations accomplishing left ventricular noncompaction criteria. Parents echocardiographic study was normal. In addition, the patient presents a normal psychomotor development. Currently the patient has three years old and is treated with enalapril and vitamin complex.

## 3. Discussion

Cardiomyopathies are a very rare disease in fetuses with a very poor outcome. Only isolated case reports and small case series were reported. In series of neonates and infants the CM occur in about 2–7%, but probably during the fetal life the prevalence is higher: 6%–11% [4].

Neonatal cardiomyopathies due to mitochondrial oxidative phosphorylation defects are severe conditions which can be either isolated or part of a multiorgan disease involving brain, skeletal muscle, liver, and kidney, in addition to heart [5].

Incidence of neonatal mitochondrial cardiomyopathies can only be approached using the reports of referral diagnostic centers. Gibson et al., in a series of 32 newborns, reported 29% of mitochondrial diseases with a neonatal onset [6]. García-Cazorla et al., in a series of 57 newborns, report a much lower proportion, 5,2% [7].

Cardiomyopathy is more often hypertrophic than dilated. However, it may eventually progress into a dilated form or left ventricular noncompaction cardiomyopathy. Antenatal manifestations such as fetal hypertrophic cardiomyopathy, arrhythmia, and/or hydrops have been reported [2]. In extremely severe cases, neonatal cardiogenic shock may occur [2].

Cardiomyopathy pathophysiological mechanisms are complex, and neonatal myocardium requires a normal mitochondrial functioning for its metabolic shift from prenatal anaerobic glycolysis to postnatal metabolism, relying on fatty acid oxidation, ketone body catabolism, and oxidative phosphorylation [8]. Complex I deficiency is regarded as the most common defect associated with cardiomyopathy [9]. Complex I is the first, largest, and most complicated respiratory complex in mitochondria. Alterations at this level can cause malfunction of a mitochondrial targeting signal and eventually lead to human diseases. Infants and children carrying complex I deficiency can develop primary lactic acidosis, which may lead to severe microcephaly and cerebral atrophy and is associated with early-onset hypertrophic cardiomyopathy and encephalopathy [1]. Biopsies from skeletal muscle, liver or heart can be performed in order to confirm the disease [10].

In neonatal cardiomyopathy, EKG monitoring is necessary for early detection of ventricular arrhythmias, which are sometimes precursors of episodes of sudden death [2]. Intensive care is often required for these severe diseases to survive the neonatal period, as many patients need ventilatory and circulatory support [2].

Mitochondrial cardiomyopathies are more severe than mitochondrial diseases without heart involvement, being the survival rate at 16 years of age for cardiomyopathic patients 18% versus 95% survival at the same age in non-cardiomyopathic patients [11].

Although heart dysfunction may be not progressive or even regress afterwards, neonatal mitochondrial cardiomyopathies often have a rapidly fatal outcome. Prenatal diagnosis and fetal echocardiography may improve perinatal care based on the expected natural history and postnatal course. In addition, detailed evaluation of fetal and maternal condition and standardized diagnostic procedures provide prognostic information for genetic counseling and further potential prenatal diagnosis.
